# A Novel Homozygous Mutation of the Acid-Labile Subunit *(IGFALS)* Gene in a Male Adolescent

**DOI:** 10.4274/jcrpe.galenos.2019.2018.0301

**Published:** 2019-11-22

**Authors:** Şükran Poyrazoğlu, Vivian Hwa, Firdevs Baş, Andrew Dauber, Ron Rosenfeld, Feyza Darendeliler

**Affiliations:** 1 İstanbul University İstanbul Faculty of Medicine, Unit of Pediatric Endocrinology, İstanbul, Turkey; 2 Cincinnati Children’s Hospital Medical Center, University of Cincinnati College of Medicine, Cincinnati Center for Growth Disorders, Cincinnati, Division of Endocrinology, Ohio, USA; 3 Children’s National Healthy System, Division of Endocrinology, Washington, USA

**Keywords:** Short stature, acid-labile subunit deficiency, IGFALS gene mutation, primary IGF-1 deficiency

## Abstract

Acid-labile subunit (ALS) forms ternary complexes with insulin like growth factor-1 (IGF-1) and IGF-binding protein-3 (IGFBP-3) and is essential for normal circulating IGF-1 levels. The* IGFALS* gene encodes the ALS and mutations in *IGFALS* cause ALS deficiency. We describe a patient with ALS deficiency with a novel homozygous frameshift mutation in *IGFALS* presenting with short stature and delayed puberty but ultimately achieving an adult height (AH) comparable to his target height (TH). A 15.25 year old boy presented with short stature (149.9 cm, -3.04 standard deviation score). The patient had a low circulating IGF-1 concentration, extremely low IGFBP-3 concentration, insulin resistance and osteopenia. The peak growth hormone (GH) response to GH stimulation test was high (31.6 ng/mL). Sequencing of *IGFALS* revealed a novel, homozygous, frameshift mutation (p.Ser555Thrfs.19). His mother and elder sister were heterozygous carriers. Although he had delayed puberty and short stature at the onset of puberty, he reached his TH and an AH similar to those of his heterozygous mother and sister. The heterozygous carriers had normal or low IGF-1 concentrations and low IGFBP-3 concentrations but not as markedly low as that of the patient. They had normally timed puberty, insulin metabolism and bone mineral density (BMD). The phenotype of ALS deficiency is quite variable. Despite short stature and delayed puberty, patients can achieve normal pubertal growth and AH. ALS deficiency may cause osteopenia and hyperinsulinemia. Heterozygous carriers may have normal prenatal growth, puberty, insulin metabolism and BMD.

**What is already known on this topic?**

Patients with acid-labile subunit (ALS) *(IGFALS)* mutations have markedly decreased insulin like growth factor-1 (IGF-1), and extremely low IGF-binding protein-3 levels. Although patients with ALS deficiency show moderate short stature, the phenotype of ALS deficiency is quite variable. Microcephaly, delay in puberty, insulin resistance and reduced bone mineral density (BMD) have been shown in some patients.

**What this study adds?**

A novel homozygous frameshift mutation in *IGFALS* (p.Ser555Thrfs.19) causes short stature and delayed puberty but ultimately, with obvious pubertal growth acceleration and good pubertal height gain, resulting in a normal adult height, comparable to the target height. Heterozygous carriers of this mutation have normal prenatal growth, puberty, insulin metabolism and BMD.

## Introduction

The majority of circulating insulin like growth factor-1 (IGF-1) is bound to IGF-binding proteins (IGFBP), mainly to IGFBP-3 and the acid-labile subunit (ALS). ALS has a major role in stabilizing the 150-kDa ternary complex. The ternary complex extends the half-life of IGF-1 from 10 minutes in the free form to more than 12 hours (1,2). Therefore, ALS is necessary to maintain normal circulating IGF-1 and IGFBP-3 levels. Patients with ALS gene (*IGFALS*) mutations have markedly decreased IGF-1 and extremely low IGFBP-3 concentrations, associated with normal or compensatory elevated growth hormone (GH) levels (3).

Patients typically show moderate short stature in contrast to other, more severe causes of primary IGF-1 deficiency. In addition to short stature, some other features have been reported in the phenotype of ALS deficiency. Although some of the clinical and laboratory features of these patients remain controversial, microcephaly, delay in puberty, insulin resistance and reduced bone mineral density (BMD) have been shown in some patients (4,5,6,7,8,9,10,11,12,13,14,15).

*IGFALS *is located on chromosome 16p13.3 and encodes the 85-kDa ALS glycoprotein. ALS is produced by the liver under GH stimulation (1,3). Homozygous or compound heterozygous mutations in *IGFALS* lead to ALS deficiency. *IGFALS *consists of two exons. To date, at least 22 different inactivating mutations of *IGFALS *have been reported (4,5,6,7,8,9,10,11,12,13,14,15). Similar to patients with homozygous or compound heterozygous mutations, heterozygous carriers were reported to be shorter than wild-type carriers (5,9,16,17).

We report the genotype and phenotype of a patient with ALS deficiency with a novel, homozygous, frameshift mutation in *IGFALS*, presenting with short stature and reaching an adult height (AH) similar to that of heterozygous carriers of this mutation.

## Methods

### Molecular Studies

Informed consent was obtained from the patient and his sister and mother. The genetic analyses were performed at the Cincinnati Center for Growth Disorders, Cincinnati Children’s Hospital Medical Center. Genomic DNA was extracted from peripheral blood leukocytes.

### Auxology

Height and weight were measured using a Harpenden stadiometer and electronic scale respectively and head circumference (HC) with a tape measure. Small for gestational age (SGA) was defined as birth weight and/or length standard deviation (SD) score (SDS) <-2.0. SDS for height, weight, sitting height/height SDS and HC calculated according to Turkish standards (18,19). Target height (TH) was calculated using the following equation TH=[father’s height (cm) + mother’s height (cm)]/2 -6.5 cm (girls) or +6.5 cm (boys) (20). The onset of puberty was defined according to Tanner standards as attainment of testicular volume ≥4 mL in boys (21). Bone age was estimated by the Greulich and Pyle method and height prediction was calculated by Bayley-Pinneau method (22).

### Serum Hormone Assays

Serum concentrations of IGF-1 and IGFBP-3 were measured by an automated immunochemiluminescence assay (Immulite 2000 XPi; Siemens Medical Solutions Diagnostics, Erlangen, Germany).

## Case Report

A 15.25 year old boy was referred to the pediatric endocrinology clinic for evaluation of short stature. His height was 149.9 cm (-3.04 SDS) and his weight 52.3 kg (-2.3 SDS) with a HC 53.8 cm (-2.0 SDS) at presentation. He was 2.05 SD shorter than his TH. Clinical and laboratory characteristics of the patient at presentation and during follow-up are given in Table 1.

At presentation, there were no dysmorphic features noted and no body disproportion. He was prepubertal; his testicular volumes were 3 mL bilaterally. He was born SGA at 40 weeks of gestation, with a weight of 2400 g (-2.7 SDS). Neuromotor development was normal. His medical history was otherwise unremarkable.

His parents were unrelated but originated from the same village. Table 2 shows clinical and laboratory characteristics of the patient and his mother and sister. Mother’s height was 155.6 cm (-1.28 SDS). She reported achieving menarche at 13 years. Father passed away due to chronic renal failure. His reported height was approximately 170 cm (-0.9 SDS). There was no information on the father’s pubertal history. TH of the patient was 169.3 cm (-0.99 SDS). His elder sister’s birth weight was 3000 g at 40 weeks of gestation (-0.9 SDS) and height was 157.1 cm (-1.02 SDS) at 21 years of age. Her age at menarche was 12 years.

Serum IGF-1 concentration of the patient was markedly reduced at 68.6 ng/mL (normal: 193-731 ng/mL). IGFBP-3 concentration was extremely low at <0.5 ng/mL (normal: 3.2-8.7 ng/mL). Thyroid function was normal. The peak GH response to GH stimulation test was high at 31.6 ng/mL. Bone age was 13.5 years at presentation. IGF generation test showed a response of serum IGF-1 from 58.4 ng/mL to 100 ng/mL, but no response of serum IGFBP-3 which did not change from baseline of <0.5 ng/mL.

During follow-up, fasting glucose concentrations of the patient were within the normal range. An oral glucose tolerance test, performed when he was 20.1 years old, showed insulin resistance (Table 2). He did not have bone pain or any fractures. His spine (L1-L4) BMD, determined by DXA at the age of 20.1 years was -3.5 SDS, indicating osteoporosis. Serum calcium, phosphate, alkaline phosphatase, parathyroid hormone and 25-OH vitamin D levels were normal.

**Figure 1:**
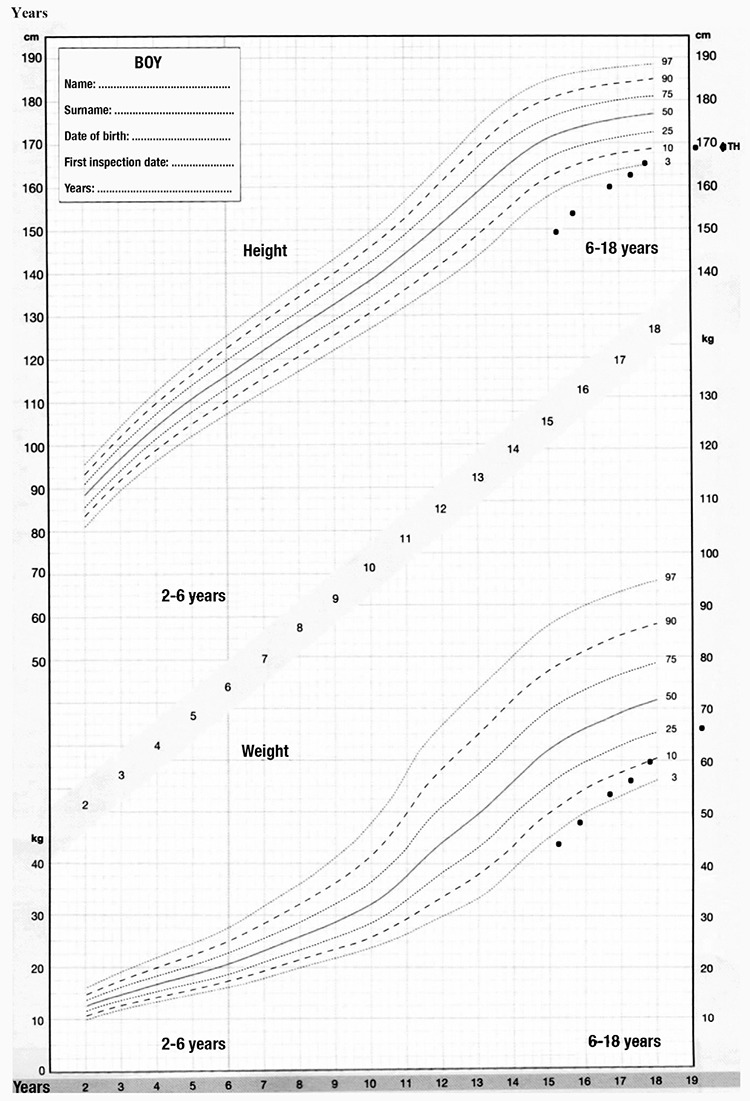
Growth chart of patient for height (at the upper panel) and weight (at the lower panel) plotted on growth chart for Turkish children (18)

Puberty of the patient started at 15.9 years. Testes volumes were 6 mL/6 mL (Tanner stage G2). His height was -3.0 SDS at onset of puberty. His bone age was retarded by approximately two years when compared to his chronological age. His peak height velocity was 7 cm/year during progression of puberty (Tanner stage G3; Figure 1) and total height gain during puberty was 19.6 cm.

At his last follow up visit at age 20.1 years, his height SDS, weight SDS and HC SDS values were -1.08, -0.31 and -1.0, respectively. He reached his TH (Table 1). Puberty was completed and testicular volumes were 20/20 mL at age 20.1 years. His bone age was 18 years. Serum LH and FSH concentrations were 2.77 mIU/mL (normal: 1.7-15.3) and 3.88 mIU/mL (normal: 1.5-12.4), respectively, with a testosterone level of 5.17 ng/mL (normal: 2.18-9.06 ng/mL).

His sister’s IGF-1 concentration was low but his mother’s was normal. Although their IGFBP-3 concentrations were decreased they were higher than that of the patient. His sister and mother had normal fasting insulin and glucose levels. Their L1-L4 BMDs were also normal (Table 2).

**Figure 2:**
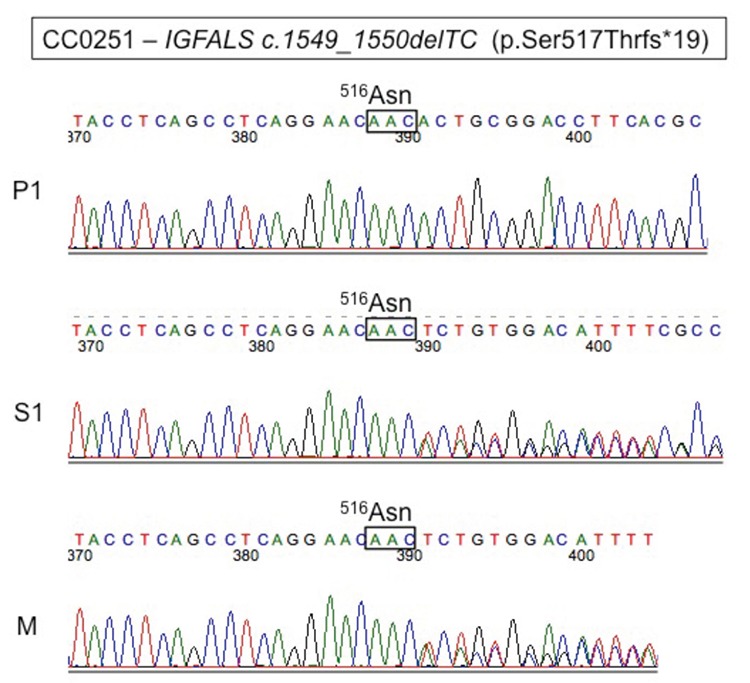
Sequence chromatograms for patient (P), his sister (S) and mother (M). The two nucleotide deletions are located immediately downstream of boxed triplex. The patient is homozygous. His sister and mother are heterozygous

Sequencing of the *IGFALS *gene revealed a novel homozygous mutation in exon 2 at c.1663-1664delTC (p.Ser555Thrfs.19). This frameshift point mutation resulted in a substitution of a serine for a threonine at position 555 of the protein leading to an early stop codon, 19 codons later (Figure 2). His mother and elder sister were heterozygous mutation carriers.

## Discussion

In our patient, low IGF-1, an extremely low IGFBP-3 concentration and moderate short stature at presentation pointed to the possibility of ALS deficiency. Molecular genetics analysis for *IGFALS *revealed a homozygous mutation in exon 2 (c.1663-1664delTC and p.Ser555Thrfs.19). The mutation is located towards the last third of the ALS protein, within LRR 20 (leucine rich repeat). The LRRs would be replaced by 19 new amino acids. These LRRs are critical to the interaction between ALS and IGFBP-3 (23,24). The majority of mutations in *IGFALS* gene result in defects within the LRR region of the protein (3). Different mutations (missense, deletion and insertion) in the ALS protein have been reported and all of the mutations reported to date are located in exon 2. This frameshift mutation in our patient is predicted to cause early protein termination and likely destabilize ALS, thus leading to nonsense-mediated decay of the truncated mRNA, resulting in ALS deficiency in our patient. Serum IGF-1 and IGFBP-3 concentrations were not so profoundly low in family members who were heterozygous carriers, similar to other heterozygous carriers reported previously (9,16,17).

In keeping with most reported cases, the main clinical feature of our patient is moderate short stature before puberty. His height SDS at presentation (-3.04 SDS) is also consistent with previous reports in ALS deficient individuals (16). During follow-up, our patient showed a normal growth pattern in puberty and reached his TH, which contrasts with most of the cases reported in the literature in which AH was approximately 1.3 to 1.5 SD below their TH SDS (4,5,6,10). Phenotypic variations between patients who are homozygous for *IGFALS *mutation have been reported. Even a degree of phenotypic variation between two siblings was demonstrated (12). Schreiner et al (8) reported an ALS deficient patient with normal height (-0.19 SDS) and growth pattern with a difference of approximately 0.5 SDS between AH SDS and TH SDS. Although van Duyvenvoorde et al (5) reported that the sitting height/height ratio was in the upper normal range in most cases, sitting height/height ratio of our patient was normal.

AH SDS of our patient is similar to the heights of his heterozygote mother and sister. The height SDSs of heterozygous carriers in our family are approximately 1 SD lower than population mean. This is consistent with previous reports indicating that heterozygous carriers are 1.0 SDS shorter than wild type subjects (5,9,17). The heterozygous carriers have a milder phenotype compared to cases with homozygous mutations (9,12,17). It is proposed that there could be a possible gene dosage effect (9,16).

Our patient and other family members who are heterozygous carriers do not have microcephaly and their HC SDSs were similar. Microcephaly was previously reported in some patients but not present in other reported cases (5,6,7,9,12). It was reported that three siblings with an *IGFALS *mutation had HCs that were lower than those of heterozygous and wild-type carriers and mean HC SDS of heterozygous carriers was 0.7 SD lower than those of non-carriers. It has been speculated that microcephaly may be related to the low IGF-1 levels, due to ALS deficiency, during fetal life (5).

Our patient showed decreased BMD Z-score but BMD Z-scores of heterozygous family members were within the normal range. There was no history of bone pain or fracture. There is conflicting evidence about the prevalence of low BMD in patients with ALS deficiency (5,6,7,11,16,17).

Our patient was born SGA and his birth size was significantly smaller than his heterozygous carrier sister. Although he was SGA, he reached an AH comparable to that of his appropriate-for-gestational age sister. It has been reported that some cases with ALS deficiency are born SGA (7,11,12,15,16). The effect of being SGA on AH in these patients needs to be investigated.

Although our patient had pubertal delay, he demonstrated an obvious pubertal growth acceleration and good pubertal height gain and reached an AH comparable to his TH. The effect of ALS deficiency on pubertal development remains controversial. Age of pubertal onset and growth pattern are still unclear in these patients. Delayed puberty was reported in 50% of males with ALS deficiency, however, normal pubertal growth has been reported in some patients with ALS deficiency (4,6,8,10,11). An adolescent female was reported with a novel homozygous mutation of the *IGFALS* gene with absent pubertal growth spurt and a slow pubertal progression, despite a normal onset of puberty (7).

Our patient had insulin resistance but heterozygous carriers in this family have normal fasting glucose and insulin levels. Insulin resistance has previously been reported in some patients with ALS deficiency (4,6,11,16). It was suggested that this may be related to the increased GH levels and the low IGF-1 levels (25).

In conclusion, we report a novel *IGFALS *mutation identified in a patient with biochemical signs of ALS deficiency and short stature, born SGA, with delayed puberty but normal growth and an AH comparable to TH. It is important to know all of the phenotypic features of ALS deficiency in order to ensure proper follow up of these patients. Although heterozygosity for ALS affects height, it seems to show no effect on prenatal growth, puberty, insulin metabolism and BMD.

## Figures and Tables

**Table 1:**
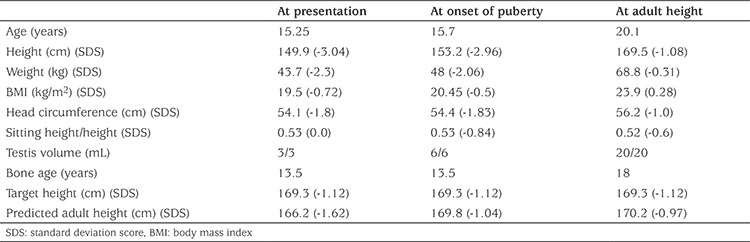
Clinical characteristics of the patient at presentation and during follow-up

**Table 2:**
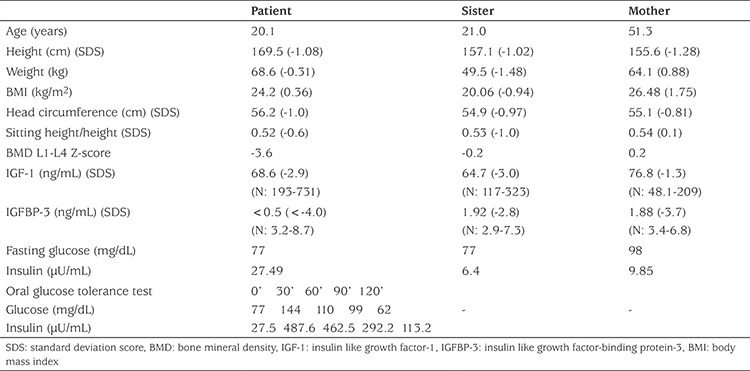
Clinical and laboratory characteristics of the patient and heterozygous carriers at last visit
